# Dissemination and Implementation Theories, Models, or Frameworks Utilized in International Aging Research: A Citation Analysis

**DOI:** 10.1007/s43477-025-00204-3

**Published:** 2026-01-06

**Authors:** Jessica Barth, Heather W. Davila, Kelly A. O’Malley, Marlena Shin, Anna Rae L. Montano, Rebecca J. Howe, A. Alex Levine, Emily Evans, Camilla B. Pimentel, Jennifer L. Sullivan

**Affiliations:** 1 Transformative Health Systems Research to Improve Veteran Equity and Independence (THRIVE) Center of Innovation (COIN), United States Department of Veterans Affairs, Providence, RI USA; 2 Department of Health Services, Practice, and Policy, Brown University School of Public Health, Brown University, Providence, USA; 3 Center for Access and Delivery Research and Evaluation, United States Department of Veterans Affairs, Iowa City, IA USA; 4 Department of Internal Medicine, Carver College of Medicine, University of Iowa, Iowa City, USA; 5 Geriatrics and Extended Care, United States Department of Veterans Affairs, Boston, MA USA; 6 Department of Psychiatry, Harvard Medical School, Harvard University, Cambridge, USA; 7 Center for Health Optimization and Implementation Research and the New England Geriatric Research Education and Clinical Center, VA Bedford Healthcare System, United States Department of Veterans Affairs, Washington D.C., USA; 8 Inpatient Geriatric Services, Hartford Hospital, Hartford, USA; 9 Department of Health Law, Policy, and Management, Boston University School of Public Health, Boston, MA USA; 10 Department of Physical Therapy, Sargent College of Health and Rehabilitation Sciences, Boston University, Boston, USA; 11 Department of Public Health, Zuckerberg College of Health Sciences, University of Massachusetts System, Boston, USA

**Keywords:** Citation analysis, Implementation science, Theories, models and frameworks, Aging, Global health

## Abstract

Dissemination and implementation (D&I) theories, models, and frameworks (TMFs) guide the translation of evidence into practice, yet their use in international aging research remains understudied. To examine which D&I TMFs are most frequently used in international aging research and how they are applied across different settings and implementation stages. We conducted a citation review of articles citing 83 D&I TMFs identified from a public repository. Articles focusing on adults aged ≥ 60 years published through January 2022 in non-US settings were included. We assessed TMF citation patterns, application across implementation processes, application across article sections, and meaningful use. Of 451 included articles from 41 countries, most originated from: Canada (21%), England/Great Britain (17%), Australia (15%), and the Netherlands (12%), representing 69% of articles. Publications increased ten-fold from 2012 to 2021, with 49 unique TMFs utilized. Five TMFs accounted for 51% of citations: Behavior Change Wheel (16%); Consolidated Framework for Implementation Research (11%); Promoting Action on Research Implementation in Health Services (9%); Reach, Effectiveness, Adoption, Implementation, Maintenance 1.0 (9%); and Explaining Behavior Change in Evidence-Based Practice (8%). Most articles (81%) used only one TMF and 39% demonstrated meaningful use. Most studies focused on pre-implementation (41%) or implementation (46%) stages, with fewer addressing intervention adaptation (4%) or sustainment (9%). While TMF use in international aging research is growing, meaningful application remains limited. The concentration in specific countries and dominance of few frameworks reveal areas for future work. Improving TMF selection, adaptation for international contexts, and application across all implementation stages has the potential to enhance global translation of aging research into practice.

**Supplementary Information:**

The online version contains supplementary material available at 10.1007/s43477-025-00204-3.

Dissemination and implementation (D&I) science is the study of methods and strategies to promote the systematic uptake, integration, and sustained use of evidence-based interventions (EBIs) (e.g., practices, innovations, interventions) across multiple levels of the socioecological framework, including individuals, organizations, and communities, to improve the quality, effectiveness, and equity of health promotion, health services, and health care. (Tabak et al., 2012) Implementation science, a core component of D&I, focuses specifically on the uptake of EBIs into routine use. It is central to identify effective strategies for making care widely accessible, and the appropriate selection and use of theories, models, and frameworks (TMFs) in implementation studies is critical to supporting this process. The use of conceptual approaches, like TMFs, plays a key role in guiding implementation by providing a structured approach to understanding and improving the strategies and activities involved in implementing EBIs. (Wang et al., 2023) TMFs support generalization by offering shared language and constructs that aid communication, implementation strategy selection, and evaluation of implementation success. Different conceptual approaches can help to identify which EBIs might work best in specific contexts and are most relevant to unique populations, settings, and systems across the research continuum. (The Lancet Global Health, 2023; Windle et al., 2024)

Improving methods of implementation science, like TMF application, are particularly relevant as the global population ages, with projections indicating that by 2050, 38% of the world’s population will be over age 65. (Keating, 2022) This demographic shift is already impacting high-income countries and will increasingly affect low- and middle-income countries, where two-thirds of adults over 60 will reside by mid-century. (Jin et al., 2015; The World Bank, 2024) These changes present significant current and future challenges for health systems worldwide (Rudnicka et al., 2020; World Health Organization, 2022) due to potential workforce shortages, (McIsaac et al., 2024) limited care coordination, (Council of the European Union, 2021; Jazieh & Kozlakidis, 2020) and insufficient EBIs to support aging in place. (Abud et al., 2022; Dussault et al., 2010; Mwakilasa et al., 2021) Despite growing research on aging-related conditions, such as, falls and cognitive decline, substantial gaps remain in translating findings into routine care. (Bennett et al., 2019) Aging research has evolved significantly, originally focusing on biomedical aspects but now encompassing psychological, social, and environmental dimensions. (Aronson, 2020; Department of Veterans Affairs, 2022) This multidisciplinary approach recognizes aging as a complex, heterogeneous process requiring coordinated study across multiple domains. (Aronson, 2020; Campisi et al., 2019; Osborne et al., 2020; Rudnicka et al., 2020) While TMFs offer a valuable approach for organizing implementation efforts, their use and application in international aging research remains inadequately documented. (Windle et al., 2024)

TMF selection depends on project goals, multilevel context, and specific implementation challenges. (Gomes et al., 2024; McIsaac et al., 2024; Strifler et al., 2018) The selection process becomes especially complex in international contexts, where economic, social, and health system variations require thoughtful adaptation to enhance meaningful application. (Ridde et al., 2020; The Lancet Global Health, 2023) Ensuring successful implementation requires adequate capacity to translate research into practices and policies that meet the needs of aging populations worldwide. (OAlonge et al., 2019; Rudnicka et al., 2020) The appropriateness of specific evidence and research for translation must be evaluated and adapted at the local level. In aging research environments, limited attention to contextually relevant implementation strategies and infrequent use of TMFs may contribute to inconsistent uptake of innovations and missed opportunities for system-level improvements. (Windle et al., 2024)

We identified two reviews that highlight the challenges of applying implementation science methods in aging research environments (Sullivan et al., 2023; Windle et al., 2024) One previously conducted citation review examined the extent to which D&I TMFs are used and applied to advance EBIs in aging research in the United States (U.S.). (Sullivan et al., 2023) The authors found that while growing, D&I theoretical framework usage remains limited, with 50% of citations concentrated among just five frameworks, and many studies citing frameworks only in single section rather than integrating them throughout the research design and analysis. (Sullivan et al., 2023) In a second review, Windle et al. (2024) examined factors influencing the implementation of innovation in aging research environments.) Their findings emphasized the need for contextually tailored strategies but revealed that only 28% of studies incorporated a D&I TMF, and even then, often without meaningful integration. These findings underscore a persistent gap between theory and practice in aging implementation research.

While prior research examined TMF citation and application in U.S. aging research, the use of TMFs in international contexts remains less understood. (Sullivan et al., 2023; Wang et al., 2023) Given the significant differences in healthcare systems, resources, and cultural factors that influence EBI implementation globally, the goals of this study were to determine: (1) which TMFs are most frequently used in international aging research and (2) how TMFs are applied across different settings and implementation stages and processes. Understanding these patterns can inform more effective selection and application of TMFs to advance EBIs for aging populations globally.

## Methods

We used the Preferred Reporting Items for Systematic Reviews and Meta-Analyses Extension for Scoping Reviews (PRISMA-ScR) to guide this citation review. (Tricco et al., 2018) This review was prospectively registered on Open Science Framework (https://osf.io/w4zkh; Project ID: 10.17605/OSF.IO/W4ZKH).

### Study Design

We performed a citation review to provide an overview of how D&I conceptual approaches of TMFs are being used in aging research internationally, building on our previous citation review in the U.S. (Sullivan et al., 2023) Citation analysis is the study of the frequency and patterns of citations in subsequent contexts, to determine the impact and importance of scholarly work as measured by how many times an article is cited. (Nicolaisen, 2007; Smith, 1981) This citation analysis maps research citing foundational D&I TMF articles.

### Identification of TMFs and Original Publications

**Fig. 1:**
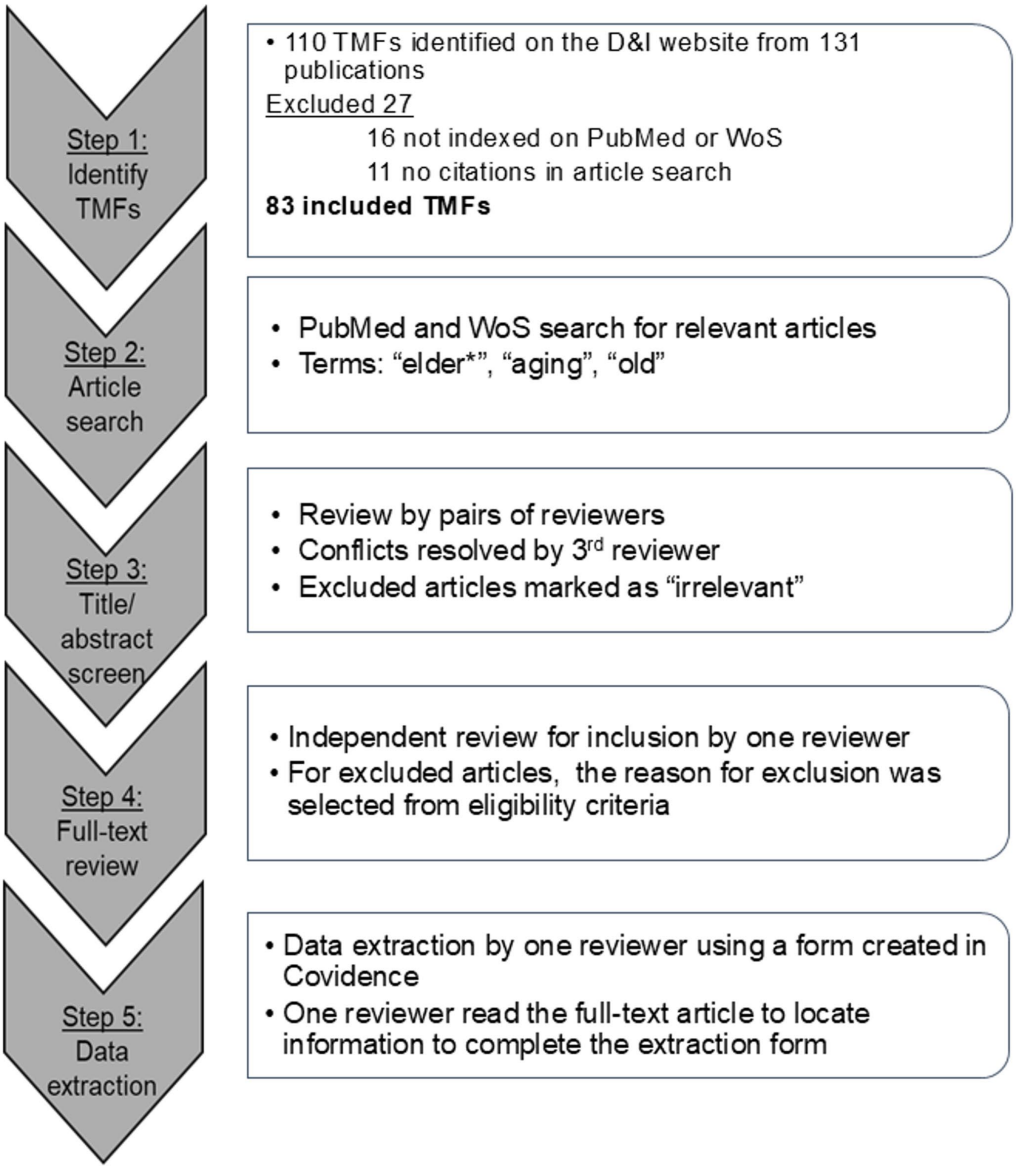
Sequential steps of the citation review

This citation review was conducted in five steps (Fig. 1). In step 1, we identified D&I TMFs from the publicly available *D&I Models in Health Research and Practice website* (https://dissemination-implementation.org/tool/explore-di-models/), a resource designed to facilitate the use of implementation science models in advancing EBIs. In January 2022, the website listed 110 TMFs with 131 associated publications that included journal articles, books, and reports. We grouped PARIHS and i-PARIHS together because i-PARIHS was not listed as a separate entry on the D&I webtool at the time of our review, whereas RE-AIM 1.0 and RE-AIM 2.0 were listed separately and thus analyzed as distinct frameworks to maintain consistency with the webtool’s categorization. Sixteen TMFs were excluded because their original publications were not indexed in PubMed or Web of Science (WoS), and 11 TMFs were removed because they had no citations. We list TMFs excluded from this review in Supplementary Table S1. We included the remaining 83 TMFs in this review.

### International Aging Research Using TMFs

In Step 2, we identified aging research articles that cited at least one of these TMFs. We searched PubMed and WoS using the terms “elder*,” “aging,” and “old” to identify articles in aging research that used at least one of the eligible TMFs. (Bakkalbasi et al., 2006) We selected these databases for their broad coverage of biomedical, behavioral, and interdisciplinary research and their capability to export citation data. We collected citations from the date of each TMFs foundational article through January 2022, and we removed duplicate citations. We uploaded the final dataset into Covidence, a systematic review software, for title and article abstract screenings and full-text reviews (Steps 3 and 4). (Veritas Health Innovation, 2023)

### Inclusion and Exclusion Criteria

We used the following criteria for both the title and abstract reviews and full-text articles reviews. We *included* articles if (a) they were published before January 28, 2022; (b) they used an eligible TMF; (c) they were written in, or translated into, English; (d) the article abstract and full text were available; (e) the study occurred exclusively in a non-U.S. country (i.e., participants/populations were recruited and/or research was carried out in a non-U.S. country); (f) the article described any stage of a research study or quality improvement (QI) project; (g) the article focused on adult participants aged > 60 years, had a mean age of adult participants of 60 years, or at least half of the sample had a mean age of 60 years; (Shenkin et al., 2017; World Health Organization, 2022) and (h) the article had an outcome tied to improving older adults’ health. We *excluded* articles if: (a) they were published after January 28, 2022; (b) they did not cite an eligible TMF; (c) they were not available in English; (d) the article abstract and/or full-text article were not available; (e) the article included a US site; (f) the article did not focus on adults > 60 years; (g) the article described nonoriginal research (e.g., protocols, reviews, commentaries, conference abstracts or dissertations); and (h) the article did not have an outcome tied to improving older adult’s health (e.g., had an educational outcome).

### Title, Abstract, and Full-Text Reviews

We screened articles for inclusion in two steps. In the title and abstract screen (Step 3), pairs of reviewers, from our study team of ten, independently reviewed titles and then abstracts of each article based on our inclusion criteria. Discrepancies between reviewers were resolved by a third reviewer. We marked articles that did not meet inclusion criteria as “irrelevant” and excluded them. Included articles moved to full-text review (Step 4). One reviewer independently reviewed each full-text article and reasons for excluding an article were documented based on eligibility criteria.

### Data Extraction of Included Articles

We did data extraction (Step 5) using a structured form developed in Covidence. The form captured the following study characteristics: country, research focus, study design, participants or population, setting, implementation stage(s) and intervention adaptation, and the cited TMF(s). (Veritas Health Innovation, 2023) To ensure consistent data collection, data extractors were oriented to the form and trained on the definitions of each field. We refined and finalized definitions through team discussions. We assessed the implementation stages of pre-implementation, implementation, and sustainment which reflect key phases in integrating EBIs into routine care and information about intervention adaptation. (Aarons et al., 2017; Chambers & Norton, 2016; Kilbourne et al., 2019; Stirman et al., 2012) A version of the data extraction form, that omits the section listing the names of the TMFs, is available in Supplemental Tables 2 and the definitions of the variable and item responses in Supplemental Table S3.

Each TMF was evaluated for its application across article sections (i.e., introduction, methods, results, discussion) and its meaningful use. Application was scored using a scale of 1 to 3, based on whether the TMF appeared in only one article section (score = 1), multiple sections (score = 2), or throughout all article sections (score = 3). (Sullivan et al., 2023) Meaningful use was defined as the TMF “guiding data collection, measurement, coding, analysis, or reporting,” and scored as a “0” if the TMF was *not* used meaningfully or a “1” if it *was* used meaningfully. (Birken et al., 2017b; Sullivan et al., 2023) We incorporated these scoring methods for both application and meaningful use of the TMF to better understand how TMFs were applied. (Sullivan et al., 2023) To ensure reliability, we conducted monthly spot checks of data extracted from the articles, which involved randomly selecting a subset of articles to verify the accuracy and consistency of the coding and data entry.

### Categorization of TMFs

We categorized each TMFs as a process model, implementation theory, or a strategy, determinant, measurement or evaluation framework with definitions from Nilsen (2015) and Wang et al. (2023). As defined by Nilsen (2015), a process model specifies steps in the process of translating research into practice, a determinant framework specifies types of barriers and enables that influence implementation outcomes, an implementation theory is developed by implementation researchers to provide understanding or explanation of aspects of implementation and an evaluation framework specifies aspects of implementation that can be evaluated to determine implementation success. Acording to Wang et al. (2023), a strategy framework is defined as the structure of the implementation interventions to facilitate the implementation process, and a measurement framework is defined as the structure of the measurement metrics of implementation constructs or influential factors.

### Analysis and Presentation of Findings

After data extraction, we exported the information captured in the data extraction forms into an Excel spreadsheet for analysis. We assessed citation trends over time using a line plot that displayed article counts by publication year, and we used a bar graph to illustrate the distribution of articles by country. We tabulated counts and percentages of study settings, participants/populations, and focus areas to provide insight into where and how TMFs were applied. We tabulated the categories of TMFs as counts and percentages, and we used a pie chart to illustrate proportion of the categories for the total sample. To analyze TMF application across implementation processes, we documented their use in three distinct stages (pre-implementation, implementation, and sustainment) and for intervention adaptation, then categorized articles by the number of implementation stages (one to three) in which they reported using TMFs. We also compared TMF application within specific article sections and meaningful use for the total sample and for categories of articles based on the number of TMFs an article used.

## Results

### Article Selection

**Fig. 2:**
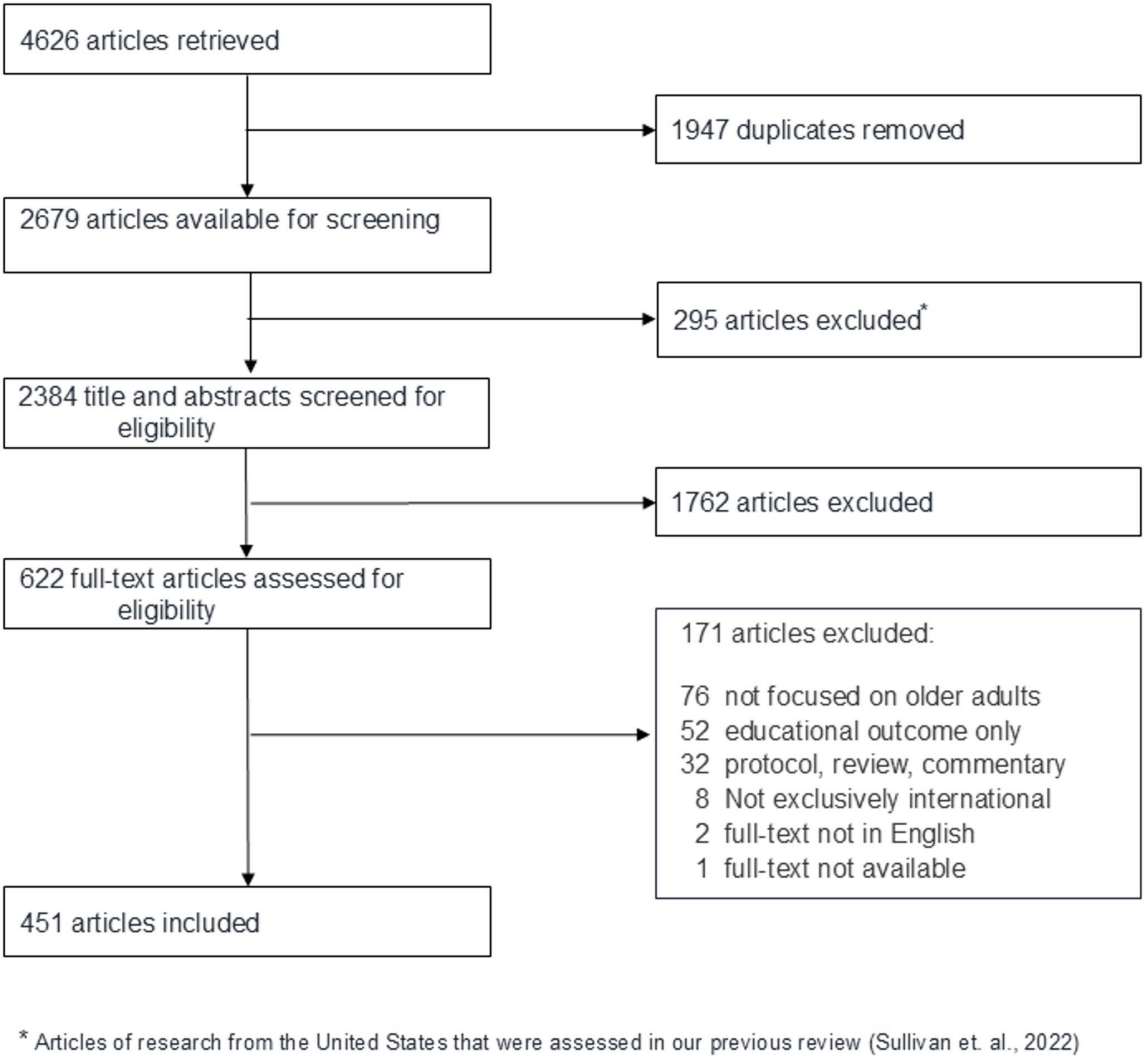
PRISMA-ScR flow diagram of article selection

Our initial search yielded 4,626 articles. We removed 1,947 duplicates. Of the 2,679 articles remaining for screening, we first removed 295 U.S.-based articles that were assessed in our previous review. (Sullivan et al., 2023) We imported the resulting 2,384 articles into Covidence. We excluded 1,762 articles in the title and abstract screen that did not meet inclusion criteria. We did full-text review of the remaining 622 articles. We excluded 171 articles, primarily because the research did not focus on older adults (*n* = 76); the outcome of the study was educational (i.e., not research or quality improvement work) (*n* = 52); or the article was a protocol, review, or commentary (*n* = 32). We present the counts of articles assessed at each step in a PRISMA-ScR flow diagram (Fig. 2). (Skolarus et al., 2017; Tricco et al., 2018) We included the remaining 451 articles in this review.

**Fig. 3:**
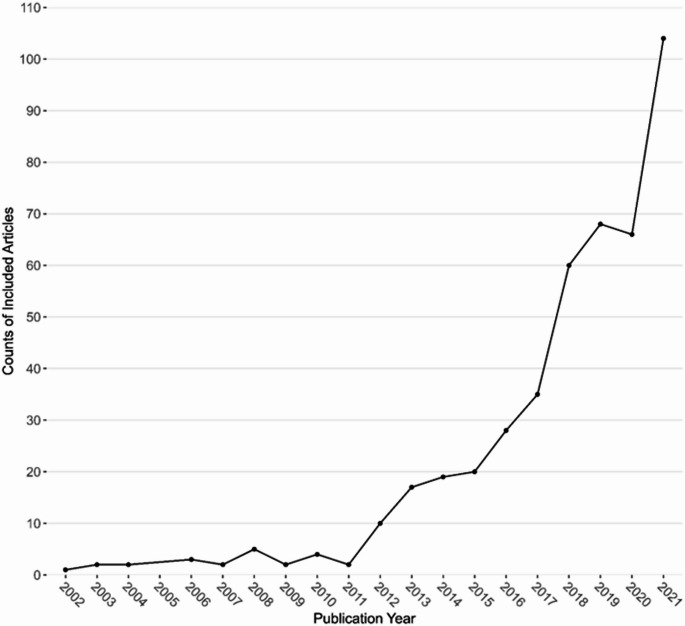
Counts or articles per year. Line graph showing the growth in articles reporting using a TMFs in aging research from 2002 to 2021. Counts of total articles (y-axis) by year (x-axis) of international aging research that used at least one TMF

Included articles were published between 2002 and December 2021. Figure 3 shows a 10-fold increase in TMF citations in international aging research articles over this period. There were fewer than five articles published annually until 2011. Between 2012 and 2019, there was a gradual increase in the number of articles published, and from 2020 to 2021, there was a sharp increase, with over 60 articles published in 2020 and over 100 published in 2021.

**Fig. 4:**
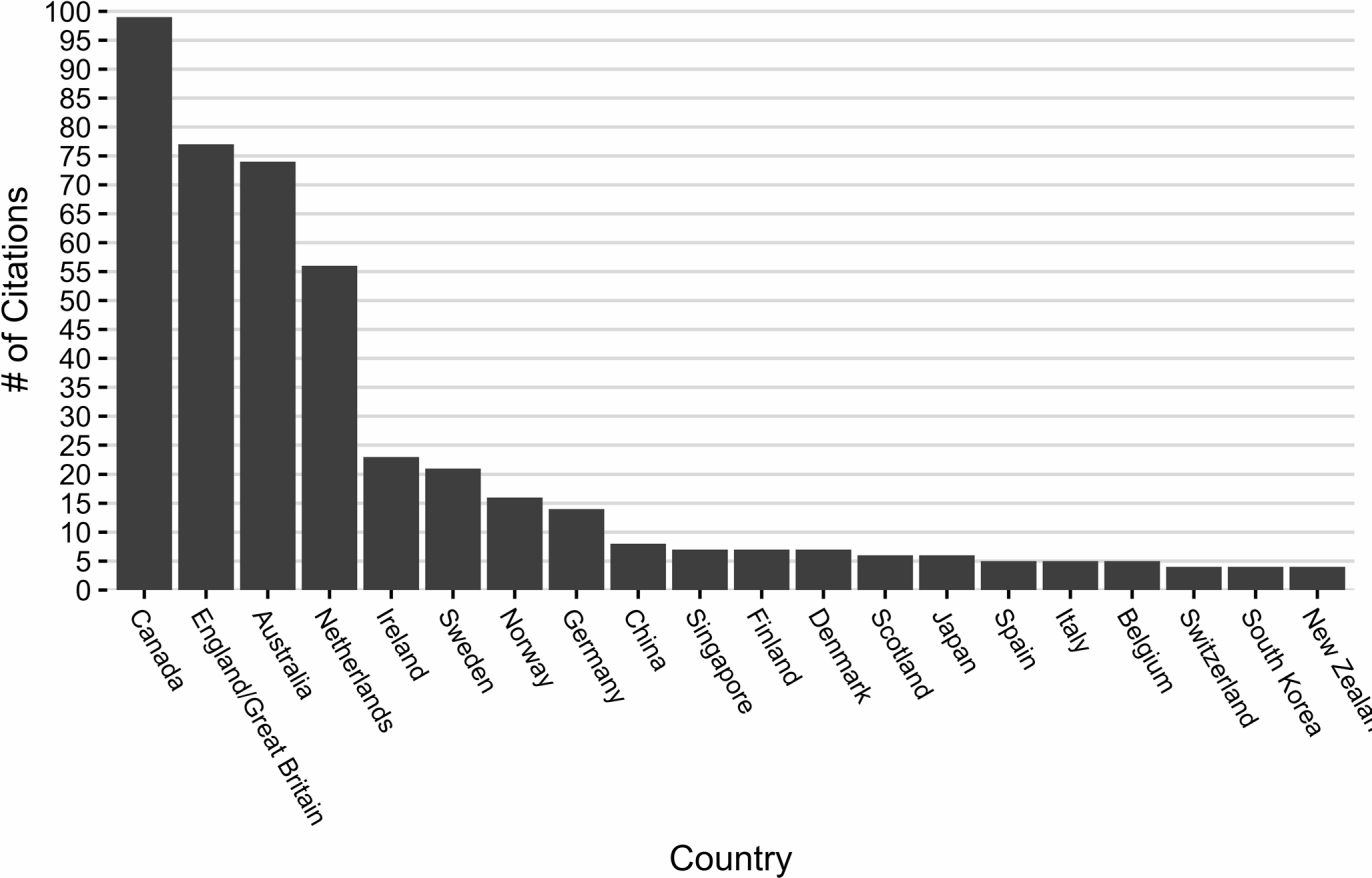
Distribution of counts of articles by country for country. Horizontal bar graph showing the distribution of article counts by country for countries that had three or more articles. The countries with over 69% of the articles were Canada (21%), England/Great Britian (17%), Australia (15%), and Netherlands (12%). The remaining countries had between 20 to 3 articles each. There were also articles from 21 countries which had fewer than 3 articles each that are not shown in the figure

Forty-one countries were represented in the articles. We show the distribution of articles by country in a bar graph for countries that had a minimum of three articles (Fig. 4). More than half of the articles (69%) were from four countries: Canada (21%), England/Great Britain (17%), Australia (15%), and the Netherlands (12%). Sixteen countries represented 30% of articles, and the remaining 1% of articles came from 21 countries that are not presented in the figure: Abu Dhabi, Belgium, Brazil, Brussels, Chile, Fiji, France, Ghana, India, Israel, Malaysia, Poland, Portugal, Saudi Arabia, Switzerland, Taiwan, Tasmania, Thailand, Turkey, Vietnam, and Wales.

### Identified TMFs

Five TMFs accounted for more than half of those cited in the articles. In Table 1 we show the count and percentage of citations for each TMF. We found that 49 of the 83 TMFs (59%, *n* = 49) were reported in 451 articles, with a total of 575 TMF citations. The five most frequently used TMFs were two behavior change models/strategy frameworks (BCW and Explaining Behavior Change in EBP), two determinant frameworks (CFIR and PARIHS), and one planning/evaluation framework (RE-AIM 1.0). (Damschroder et al., 2009; Glasgow et al., 1999; Harvey & Kitson, 2016; Kitson et al., 1998; Kitson et al., 2008; Michie et al., 2014; Michie et al., 2005; Michie et al., 2011; Rycroft-Malone, 2004) More than half of the articles (64%, *n* = 365) used a single TMF and the highest number used in one article was five. (Gray et al., 2020) A total of 87 articles out of the 451 (20%) reported using two, three, four, or five TMFs. Of these articles, most reported using two (68%, *n* = 60) or three (24%, *n* = 21) TMFs and very few reported using four (8%, *n* = 7) or five (< 1%, *n* = 1). In Supplemental Table S4 we show the combinations of TMFs reported in the 87 articles that reported using more than one TMF.

**Table 1:** Count and percent of theory, model, or framework use in included articles

Name of theory, model, or framework	Count (%)
Behavior Change Wheel (BCW)	93 (16)
Consolidated Framework for Implementation Research (CFIR)	63 (11)
Promoting Action on Research Implementation in Health Services (PARIHS)	52 (9)
RE-AIM 1.0 Framework (RE-AIM)	51 (9)
Explaining Behavior Change in Evidence-Based Practice	48 (8)
Greenhalgh Diffusion of Innovations in Service Organizations	43 (7)
Normalization Process Theory	34 (6)
Organizational Theory of Innovation Implementation	24 (4)
Proctor’s Implementation Outcomes	22 (4)
Canadian Institutes of Health Research Knowledge Translation within the Research Cycle Model or Knowledge Action Model	21 (4)
Theoretical Domains Framework	18 (3)
Dynamic Sustainability Framework	11 (2)
Intervention Mapping	9 (2)
Determinants of Innovation within Health Care Organizations	9 (2)
Interactive Systems Framework	8 (1)
Stirman framework and coding system for modifications and adaptations of evidence-based interventions	7 (1)
Davis’ Pathman-PRECEED Model	6 (1)
Replicating Effective Programs Framework	6 (1)
General theory of implementation	6 (1)
Community Based Participatory Research (CBPR)	4 (< 1)
Pronovost’s 4E’s Process Theory	4 (< 1)
Utilization-Focused Surveillance Framework	3 (< 1)
Conceptual Model of Implementation Research	3 (< 1)
Research Knowledge Infrastructure	2 (< 1)
Practical, Robust Implementation and Sustainability Model (PRISM)	2 (< 1)
Weiner organizational readiness	2 (< 1)
Knowledge Exchange Framework	2 (< 1)
RE-AIM 2.0/Contextually Expanded RE-AIM	2 (< 1)
Critical Realism & the Arts Research Utilization Model (CRARIUM)	2 (< 1)
Iowa Model of Evidence-Based Practice	1 (< 1)
Designing and evaluating interventions to eliminate racial and ethnic disparities in health care	1 (< 1)
Exploration, Preparation, Implementation, Sustainment (EPIS) model (Conceptual Model of Evidence-based Practice Implementation in Public Service Sectors)	1 (< 1)
Stetler Model of Research Utilization	1 (< 1)
“4E” Framework for Knowledge Dissemination and Utilization	1 (< 1)
Framework for Enhancing the Value of Research for Dissemination and Implementation (FRAME)	1 (< 1)
Ottawa Model of Research Use	1 (< 1)
Generic Implementation Framework	1 (< 1)
Evidence Integration Triangle	1 (< 1)
Framework for Analyzing Adoption of Complex Health Innovations	1 (< 1)
Framework for the Dissemination & Utilization of Research for Health-Care Policy & Practice	1 (< 1)
Caledonian Practice Development Model	1 (< 1)
EMTReK - Evidence-based Model for the Transfer and Exchange of Research Knowledge	1 (< 1)
Stages of Research Utilization Model	1 (< 1)
Framework for Spread	1 (< 1)
Organizational Theory of Innovation Implementation	1 (< 1)
Health Equity Implementation Framework	1 (< 1)
Policy Framework for Increasing Diffusion of Evidence-based Physical Activity Interventions	1 (< 1)

### Categorization of TMFs

**Fig. 5:**
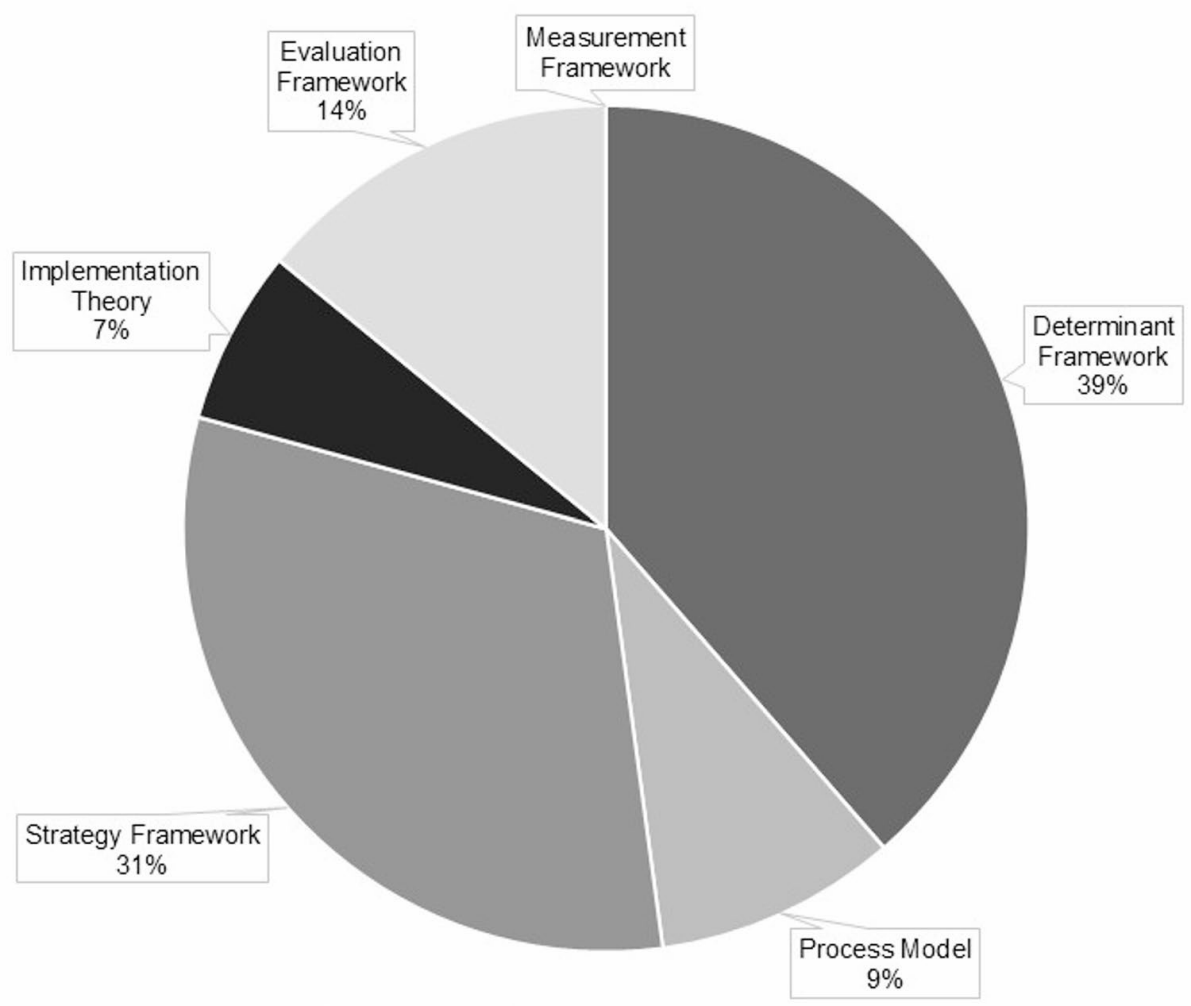
Distribution of TMF categories. Pie chart illustrates the proportional representation of different categories of TMFs identified in the articles. The chart highlights the relative prevalences of each category, within the five categories

In Fig. 5, we present a pie chart to display the proportion of the category of the TMFs relative to the total sample. Of the 585 TMFs, the most common category was determinant (*n* = 225, 38%) or strategy (*n* = 183, 31%) frameworks and the least common was measurement framework (*n* = 2, < 1%) or process model (*n* = 54, 9%). The full list of TMFs and their categorization is presented in Supplemental Table S5.

### Settings, Populations, and Implementation Processes

In Table 2 we present the counts and percentages of the different settings, populations, and areas of research extracted from the articles. The most frequently reported settings were the outpatient clinic (e.g., primary care physician office, pharmacy); community-based programs (i.e., group-based diabetes self-management program, active aging group-based counseling intervention); hospitals; and nursing homes. We also found that articles included different types of participants or populations. The most frequently reported were older adults (44%) and staff/clinical providers caring for older adults (38%). Additionally, we found variation in research focus areas within the articles. The most frequent research areas were quality of clinical care (36%); long-term services and supports (i.e., reducing falls in nursing homes) (20%); and physical activity/exercise (14%).

**Table 2:** Count and percentage of settings, populations, and focus area of research of included articles

Variable	Count (%)
*Setting(s)*	
Clinic	134 (24)
Community-based program	127 (23)
Hospital	123 (22)
Nursing home	110 (20)
Non-institutional LTSS	60 (10)
Home	5 (1)
*Participants/Population*	
Older adults	313 (44)
Staff/Clinical providers	273 (38)
Caregivers	56 (8)
Program(s)	32 (5)
Community collaborators	26 (4)
Other^*^	10 (1)
*Focus area of research*	
Quality of clinical care	244 (36)
Long-term services and supports	132 (20)
Physical activity/exercise	95 (14)
Rehabilitation	75 (11)
Quality of life	60 (9)
Mental health	45 (7)
Other^**†**^	20 (3)

^*^ Includes trainees, peer-educators, and administrators

^**†**^Includes nutrition uptake, palliative care, and oral hygiene

Articles reported using TMFs in different implementation stages (i.e., pre-implementation, implementation, and sustainment) or for intervention adaptation processes regardless of stage, with some articles reporting TMF use across multiple categories. TMFs were used most frequently in pre-implementation and implementation stages. When TMF use spanned multiple stages, it typically followed a sequential process, moving from pre-implementation to implementation to post-implementation, rather than being used in a way that blends or conflates multiple phases without clear distinction. For example, one study first applied a TMF during the implementation stage and subsequently during sustainment. (Verweij et al., 2021) Intervention adaptation was categorized in 25 articles, and all occurred in combination with one or more implementation stages, mostly the implementation phase (87%, *n* = 55).

### TMF Application and Meaningful Use

Of the 575 TMFs reported in the eligible articles, 55% (*n* = 316) were found in a single section; 29% (*n* = 169) appeared in multiple sections; and 19% (*n* = 107) were found in all sections (i.e., introduction, methods, results, analysis, discussion) of the article.

We rated meaningful use as 39% (*n* = 223) for the TMFs for the total sample. Articles citing 1 TMF had 40% meaningful use, while articles citing more than 1 TMF had an average of 32% meaningful use.

## Discussion

In this review we examined how TMFs are used and applied in international aging research to understand their role in translating evidence into practice across diverse global contexts, external to the U.S. Three key findings emerge that address our research objectives: (1) while TMFs are increasingly used in international aging research, their meaningful application varies considerably; (2) a small subset of TMFs dominates the literature, with five frameworks accounting for over half of all citations; and (3) TMFs are primarily used in pre-implementation and implementation stages, with less of a focus on sustainment and/or intervention adaptation. These findings highlight both the progress made in implementation science for aging research globally and the significant opportunities to apply TMFs to enhance the systematic deployment of evidence-based approaches across diverse contexts and throughout the full implementation continuum.

Our findings reveal a significant uptake of use of TMFs in international aging research, with a 10-fold increase in articles citing TMF use between 2002 and 2021. This suggests a growing recognition of implementation science’s value in this field. While research from 41 countries was included in our review, the concentration of this growth was in high-income countries, with 69% of articles originating from studies conducted in Canada, England/Great Britain, Australia, and the Netherlands. This pattern may suggest potential disparities in implementation science capacity, (World Health Organization, 2022) and specifically, may reflect established research infrastructure and healthcare funding mechanisms in these countries, particularly with nationalized health care systems that facilitate comprehensive aging services. (Aronson, 2020; Teasell et al., 2025; World Health Organization, 2022) Efforts have begun to understand how TMFs are used or specific models adapted (e.g., CFIR and RE-AIM) for use in low- and middle-income countries, (Evans et al., 2022; Lee et al., 2017; Means et al., 2020; Quinn et al., 2019). Future research can expand these efforts to specifically examine how TMFs are adapted and applied in aging research from low- and middle-income countries to identify effective strategies for implementing evidence-based approaches in diverse healthcare contexts with limited resources.

The distribution of research settings in our review, with nearly equal representation across clinics (24%), community-based programs (23%), hospitals (22%), and nursing homes (20%), demonstrates that TMFs are being applied across the continuum of care for older adults. The frequent focus on clinical care quality (36%) and long-term services and supports (20%) reflects the global urgency of improving healthcare and expanding aging-related care infrastructures. (Abud et al., 2022; Beech et al., 2017; Bennett et al., 2019; Friedman et al., 2024; Harasym et al., 2020) However, areas such as geriatric mental health (7%) remain underrepresented, revealing a need to broaden implementation science applications to the full spectrum of aging-related needs. (Le et al., 2022)

We found only a small subset of TMFs accounted for most usage, with BCW (16%), CFIR (11%), PARIHS (9%), RE-AIM 1.0 (9%), and Explaining Behavior Change in EBP (8%) comprising over half of all TMF citations. While their prevalence reflects their foundational role in the field, (Glasgow et al., 2019; Kirk et al., 2016; Mrklas et al., 2022; Sullivan et al., 2023; Wang et al., 2023) it may overshadow lesser known but potentially more contextually appropriate frameworks for aging research depending on the purpose of the work. (Armstrong et al., 2006; Brachaniec et al., 2006; Elliott et al., 2003; Farkas & Anthony, 2007; Farkas et al., 2003; Riley et al., 2009) Researchers may default to these familiar TMFs, despite others potentially being a better fit for study objectives, settings, or stages. (Strifler et al., 2018) This underscores the need for thoughtful TMF selection and possible use of complementary or adapted frameworks for aging-specific contexts, (Strifler et al., 2020) such as, the new features included in the D&I TMF webtool, (Gomes et al., 2024; Rabin et al., 2025) the Theory Comparison and Selection Tool (T-CaST), (Birken et al., 2018) or the Systematic Evaluation and Selection of Implementation Science Theories, Models, and Frameworks (SELECT-IT). (Fontaine et al., 2025),

Aging specific TMFs, such as “4Es Framework”, (Farkas et al., 2003) Tailored Implementation for Chronic Diseases (TICD), (Wensing, 2017; Wensing et al., 2011), and Evidence-based Model for the Transfer and Exchange of Research Knowledge (EMTReK), (Prihodova et al., 2015) offer constructs that may be underrepresented in more commonly used models. For example, TICD includes domains such as guideline factors, individual health professional characteristics, and patient-specific considerations, elements particularly relevant in aging populations where multimorbidity, functional limitations, and care transitions are common. Similarly, eMTReK emphasizes system-level transitions and coordination of care, which are critical in geriatric settings. While these frameworks often overlap with more widely used TMFs, their unique constructs may better support implementation efforts in aging research and clinical quality improvement. Greater awareness and accessibility of these frameworks could help researchers align TMF selection more closely with the specific needs of aging populations.

Despite increasing publication trends, more than half of studies referenced TMFs in only one section, suggesting superficial use, primarily to justify implementation efforts rather than shape design, data collection, or evaluation. (Birken et al., 2017a; Birken et al., 2018; Mrklas et al., 2022; Smith et al., 2024; Wang et al., 2023; Windle et al., 2024) Only 39% of TMFs were meaningfully applied, pointing to a missed opportunity to enhance methodological rigor. This aligns with prior findings citing a lack of practical guidance, overwhelming TMF proliferation, and limited training, particularly in aging research environments, as barriers to effective TMF use. (Windle et al., 2024) Educational initiatives to build capacity regarding practical application of TMFs across the research lifecycle and improve measurement of meaningful use would help bridge this gap. (Gomes et al., 2024; Kilbourne et al., 2019; Lynch et al., 2018; Tabak et al., 2012; Wang et al., 2023; Windle et al., 2024)

Our review also found limited application of TMFs to later implementation stages and processes: only 13% of studies addressed sustainment and 4% focused on intervention adaptation. (Aarons et al., 2017; Miller et al., 2021) This gap may stem from the early maturity of many implementation projects or the tendency to focus on discrete implementation phases. Additionally, the typical 3- to 5-year funding timelines for implementation research may constrain the ability to meaningfully apply or evaluate TMFs in the sustainment phase. (Kepper et al., 2024) Considering sustainment earlier in the research process and identifying mechanisms to support long-term follow-up, such as supplemental funding or integration into larger programs of research, may help address this limitation. For example, programs like Veterans Affairs (VA) Quality Enhancement Research Initiative (QUERI), which operates within a sustained infrastructure, offers a model for embedding sustainment into long-term implementation strategies. (Braganza & Kilbourne, 2021; Demakis et al., 2000; Kilbourne et al., 2019) Early engagement with healthcare systems may also create opportunities for co-funding or internal support for sustainment activities, particularly within learning health systems where sustainability can be embedded into routine operations. (Braganza et al., 2022; Chambers & Norton, 2016; McDonald et al., 2022; Yakovchenko et al., 2023)

A lack of attention to later stages poses a risk to long-term impact, especially in under-resourced settings where sustainability is critical. TMFs such as the Framework for Reporting Adaptations and Modifications to Evidence-based Interventions (FRAME), which explicitly address adaptation processes, should be prioritized to better understand how EBIs can evolve and be maintained in diverse contexts. (Escoffery et al., 2019; Park, 2010; Stirman et al., 2019) The D&I TMF webtool now includes sustainability as a key construct, enabling users to filter TMFs that address the sustainment of EBIs. New tools, such as the Designing for Dissemination & Sustainability Action Planner (D4DS), are also emerging to support collaborative planning and enhance EBI impact. (Kepper et al., 2024) However, the field still needs clear guidance and decision-support tools to help researchers select or adapt TMFs for long-term implementation, which could improve the stability and scalability of innovations. (Bakkalbasi et al., 2006; Kilbourne et al., 2019; Rabin et al., 2018; Shelton et al., 2018; Stirman et al., 2012; Strifler et al., 2018; Windle et al., 2024)

Finally, while this review centered on international research, a prior citation review of U.S. aging studies found similar patterns (Supplemental Figs. 1 A and 1B). (Sullivan et al., 2023) Both reviews observed increasing use of TMFs over time and a dominance of five frameworks, particularly CFIR and RE-AIM, across regions. Despite different growth rates, (nearly ten-fold increase internationally vs. two- to three-fold in the U.S.) both found limited attention to sustainment, highlighting the global need to improve TMF selection, integration, and training to support sustained and adaptable implementation efforts in aging research. (Bennett et al., 2019; Glasgow et al., 2019; Kirk et al., 2016; Mrklas et al., 2022; Sullivan et al., 2023; Wang et al., 2023; Windle et al., 2024)

This review provides a novel contribution by systematically examining how TMFs are used and applied in international aging research. Key strengths include a rigorous, systematic citation analysis methodology, broad inclusion of research across multiple disciplines and global contexts, building on prior research to identify key trends in TMF use and application patterns, (Gustafson et al., 2023; Smith et al., 2024; Sullivan et al., 2023; Wang et al., 2023) and systematic comparison with U.S. TMF use patterns.

However, several limitations should be considered when interpreting our findings. First, we used the World Health Organizations (WHO) definition of older people as those aged 60 years and older. (World Health Organization, 2022) However, across the 41 countries in this review, it is possible that older adults may be defined with different age criteria. (Shenkin et al., 2017) Second, our review was restricted to English-language publications, creating a sampling bias against non-English speaking authors. Additionally, the D&I website from which we identified TMFs only included English-language frameworks, potentially overlooking valuable TMFs developed and published in other languages. (Tabak et al., 2012) While English serves as the predominant language in scientific publishing, particularly among most high-impact journals, these language limitations likely reduced representation from non-English speaking regions. (Teasell et al., 2025)

Third, our methodological decisions may have systematically excluded research from low- and middle-income countries. By excluding preprints and articles with U.S. author groups, we potentially missed low- and middle-income country research since: (1) research more frequently appears as preprints but has lower conversion rates to peer-reviewed publications; (Eckmann & Bandrowski, 2023; Harris et al., 2017; Lund, 2022; Muula, 2008) (2) researchers often collaborate with U.S. colleagues to access resources and improve publication chances; (Chetwood et al., 2015; Eckmann & Bandrowski, 2023) and (3) the structural challenges in low- and middle-income research environments, including resource limitations and institutional instability, reduce publication rates. (Harris et al., 2017; Lund, 2022; Muula, 2008) Fourth, our reliance on PubMed and WoS databases likely introduced additional bias against low- and middle-income country research, as these databases favor established, high-income country journals while newer journals (which often have lower publication costs and more low- and middle-income country research content) are less frequently indexed. (Eckmann & Bandrowski, 2023)

Fifth, the TMFs were categorized using methods originally described by Nilsen (2015) and later adapted by Wang et al. (2023). However not all TMFs included in this review had been previously categorized in those works. Of the 49 TMFs, 47% (*n* = 23) had not been categorized before (see Supplemental Table S5). This highlights the need for the field to evaluate the value of categorizing TMFs and to establish a consensus on both the rationale and methodology for doing so by model type. Finally, citation analysis methods cannot capture qualitative insights into why specific TMFs were selected or how they were applied across different contexts, which limits our understanding of the decision-making processes behind TMF use. Additionally, because the first step of our citation analysis involved identifying seminal TMF publications listed on the D&I website, we excluded TMFs that were only published in books or book chapters and not indexed in PubMed or Web of Science. As a result, we may have missed aging-related research that cited TMFs, such as Diffusion of Innovations, if those citations were not captured in the databases we used. (Greenhalgh et al., 2004)

This review demonstrates increasing international use of TMFs in advancing EBIs for global aging populations, while also revealing important gaps in how TMFs are applied. The concentration of publications from specific countries, dominance of a small subset of frameworks, limited meaningful application of TMFs, and insufficient focus of use of TMFs in the sustainment stage and intervention adaptation represent areas for future development. As aging populations increase, healthcare systems will face increasing pressure to deliver effective, evidence-based care to this population. The field’s continued development will require careful attention to both universal implementation principles and local contextual factors that influence successful translation of evidence into practice. Future work should focus on expanding TMF use in diverse global settings and understanding how best to use TMFs in implementation science projects to maximize the translation of aging research into effective practice across healthcare contexts and systems.

## Supplementary Information

Below is the link to the electronic supplementary material.

Supplementary Material 1

## Data Availability

The data, analytic methods, or materials are available to other researchers for replication purposes and can be accessed by contacting Dr. J. L. Sullivan at jennifer.sullivan@va.gov. The study reported in the manuscript was preregistered at Open Science Framework (https://osf.io/w4zkh; Project ID: 10.17605/OSF.IO/W4ZKH).
