# Supplementary material for: Dissemination and Implementation Theories, Models, or Frameworks Utilized in International Aging Research: A Citation Analysis
Source: Glob Implement Res Appl. 2026 Jan 6;6(2):200–16. doi: 10.1007/s43477-025-00204-3 (PMC13216148; doi:10.1007/s43477-025-00204-3)
Supplement: Supplementary file 1 — Supplementary Material 1 [file 43477_2025_204_MOESM1_ESM.docx]

**Supplemental Tables and Figures:**

**Supplemental Table S1:** Theory, model, and frameworks excluded from this review

**Supplemental Table S2.** Abbreviated version of the data extraction tool built in Covidence

**Supplemental Table S3:** Definitions and context for extracted variables in the data extraction form

**Supplemental Table S4:** Included articles that cited a combination of more than one TMF

**Supplemental Table S5:** Categories of TMFs cited in international aging research

**Supplemental Figure 1A**: Count of articles per year for international and US aging research

**Supplemental Figure 1B**: Count of articles by country

| **Supplemental Table S1.** Theory, model, and frameworks and citations excluded from this review | | | |
| --- | --- | --- | --- |
| **TMF** | **# Times Cited^1^** | **Citations** | **Exclusion Reason** |
| *TMFs not indexed on PubMed or Web of Science* | | | |
| ACE Star Model of Knowledge Transformation | 44 | Stevens, K. R. (2004). ACE Star Model of EBP: Knowledge Transformation. Academic Center for Evidence-based Practice. The University of Texas Health Science Center at San Antonio. | 1 Book |
| Active Implementation Framework | 1870 | Fixsen DL, Naoom SF, I KA, Friedman RM, Wallace F. Implementation research: a synthesis of the literature. Tampa FL: University of South Florida, Louis de la Parte Florida Mental Health Institute, The National Implementation Research Network, 2005. FMHI Publ. #231.  Institute FCD. National Implementation Research Network. 2008. | 2 Reports |
| Adaptation in dissemination and implementation science | 39 | Baumann AA, Cabassa LJ, Stirman SW. Adaptation in dissemination and implementation science: Oxford University Press; 2017. <https://doi>.org/10. 1093/oso/9780190683214.001.0001/oso-9780190683214-chapter-17. | 1 Book |
| Conduct and Utilization of Research in Nursing (CURN) | 20 | Horsley, JA.; Crane, J.; Crabtree, MK.; Wood, DJ. Chapter 1: Introduction. Using research to improve nursing practice: A guide CURN project. New York, NY: Grune & Stratton; 1983. P. 1-10. | 1 Book |
| Contextual Frameworks for Research on the Implementation of Complex System Interventions | 24 | Rojas Smith L, Ashok M, Dy SM, Wines RC, Teixeira-Poit S. Contextual Frameworks for Research on the Implementation of Complex System Interventions. Methods Research Report. (Prepared by the RTI International– University of North Carolina at Chapel Hill Evidence-based Practice Center under Contract No. 290-2007-10056-I.) AHRQ Publication. No. 14-EHC014-EF. Rockville, MD: Agency for Healthcare Research and Quality; March 2014. [www.effectivehealthcare](http://www.effectivehealthcare).ahrq.gov/reports/final.cfm. | 1 Report |
| Diffusion of Innovation | 19700 | Rogers EM. Diffusion of innovations. 5^th^ ed. New York: Free Press, 2003. | 1 Book |
| Facilitating Adoption of Best Practices (FAB) Model | 5 | Damush TMDV, Bravata DM, Plue L, Woodward-Hagg H, Williams LS. Facilitation of Best Practices (FAB) Framework. Stroke Quality Enhancement Research Initiative (QUERI) Center Annual Report presentation, 2008. | 1 Report |
| Framework for Dissemination of Evidence-Based Policy | 8 | Dodson EA, Brownson RC, Weiss SW. Policy dissemination research. In: Brownson R, Colditz G, Proctor EK, eds. Dissemination and implementation research in health: translating science to practice. Oxford, New York: Oxford University Press, 2012. | 1 Book |
| Framework for the Transfer of Patient Safety Research into Practice | 18 | Nieva VF, Murphy R, Ridley N, et al. From science to service: a framework for the transfer of patient safety. In: Advances in patient safety: from research to implementation. Vol. 2. AHRQ Publ 050021. Rockville, MD: Agency for Healthcare Research and Quality, 2005. | 1 Book |
| Interacting Elements of Integrating Science, Policy, and Practice | 1 | TIDIRH Working Group. Interacting elements of integrating science, policy, and practice. In: Training institute for dissemination and implementation research in health. Conference proceedings. Chapel Hill NC, 2011. | 1 Conference Proceeding |
| Johns Hopkins Nursing Evidence-Based Practice Model and Guidelines | 94 | Newhouse, RP.; Dearholt, SL.; Poe, SS.; Pugh, LC.; White, KM. Johns Hopkins nursing evidence based practice model and guidelines. Indianapolis: Sigma Theta Tau International Honor Society of Nursing; 2007. | 1 Book |
| Marketing and Distribution System for Public Health | 11 | Kreuter MW, Casey CM, Bernhardt JM. Enhancing dissemination though marketing and distribution systems: a vision for public health. In: Brownson RC, Colditz G, Proctor EK, eds. Dissemination and implementation research in health: translating science to practice. Oxford, New York: Oxford University Press, 2012. | 1 Book |
| Precede-Proceed Model | 5 | Green LW, Kreuter MW. Health program planning: an educational and ecological approach. 4^th^ ed. New York: McGraw-Hill, 2005. | 1 Book |
| Real-World Dissemination | 73 | Chambers D, Raingeisen H, Hoagwood K, Patel V (adapted by). Leading clinical practice change. In: Dopson S, Mark AL, eds. Leading health care organizations. Houndmills, UK: Palgrave Macmillan, 2003. Pettigrew AM, Ferlie E, McKee L. Shaping strategic change: making change in large organizations: the case of the National Health Service. Thousand Oaks CA: Sage, 1992. | 2 Books |
| Research Development Dissemination and Utilization Framework | 90 | Havelock RG. Planning for innovation through dissemination and utilization of knowledge. Ann Arbor MI: Centre for Research on Utilization of Scientific Knowledge, Institute for Social Research, University of Michigan, 1969. | 1 Book |
| Streams of Policy Process | 6110 | Kingdon JW. Agendas, alternatives, and public policies. Boston: Little, Brown, 1984. Kingdon JW. Agendas, alternatives, and public policies. Updated 2^nd^ ed. Boston: Longman, 2010. | 2 Books |
| *TMFs excluded for no citations in this review* | | | |
| Rosswurm & Larabee 'Research Utilization Model' | 152 | Kavanagh D, Connolly P, Cohen J. Promoting evidence-based practice: implementing the American Stroke Association's acute stroke program. Journal of nursing care quality. Apr-Jun 2006;21(2):135-142  Long LE, Burkett K, McGee S. Promotion of safe outcomes: Incorporating evidence into policies and procedures. The Nursing clinics of North America. Mar 2009;44(1):57-70, x-xi | |
| Effective Dissemination Strategies | 14 | Scullion PA. Effective dissemination strategies. Nurse Res 2002;10(1):65–77. | |
| Model for Improving the Dissemination of Nursing Research | 7 | Funk SG, Tornquist EM, Champagne MT. A model for improving the dissemination of nursing research. West J Nurs Res 1989;11(3):361–72." | |
| Dissemination of Evidence-based Interventions to Prevent Obesity | 27 | Dreisinger ML, Boland EM, Filler CD, Baker EA, Hessel AS, Brownson RC. Contextual factors influencing readiness for dissemination of obesity prevention programs and policies. Health Educ Res 2012;27(2):292–306. | |
| Health Promotion Technology Transfer Process | 3 | The original publication(s) of the model. Orlandi MA. Health promotion technology transfer: organizational perspectives. Can J Public Health 1996;87 (S2):S28–S33. | |
| Conceptualizing Dissemination Research and Activity: Canadian Heart Health Initiative | 9 | Riley BL, Stachenko S, Wilson E, et al. Can the Canadian Heart Health Initiative inform the population Health Intervention Research Initiative for Canada? Can J Public Health 2009;100(1S):S120–S126.  Elliott SJ, O’Loughlin J, Robinson K, et al. Conceptualizing dissemination research and activity: the case of the Canadian Heart Health Initiative. Health Educ Behav 2003;30(3):267–82; discussion 283–6. | |
| Conceptual Framework for Research Knowledge Transfer and Utilization | 17 | Kramer DM, Cole DC. Sustained, intensive engagement to promote health and safety knowledge transfer to and utilization by workplaces. Sci Commun 2003;25(1):56. | |
| Adherence Optimization Framework | 14 | Owoeye OBA, McKay CD, Verhagen EALM, et al/ Advancing adherence research in sport injury prevention British Journal of Sports Medicine 2018;52:1078-1079. | |
| Conceptual Framework For The Comparative Analysis of Policy Change | 52 | Bauer MW, Knill C. A conceptual framework for the comparative analysis of policy change: Measurement, explanation and strategies of policy dismantling. J Comp Policy Anal. 2014;16(1):28-44. | |
| Dissemination and Implementation Framework for an Early Childhood Obesity Prevention Program | 1 | Bergling, Emily, Charlotte Farewell, and Jini Puma. "Development of a Dissemination and Implementation Framework for an Early Childhood Obesity Prevention Program." Journal of Nutrition Education and Behavior 52.12 (2020): 1160-1165 | |
| Linking Systems Framework | 12 | Robinson K, Elliott SJ, Driedger SM, et al. Using linking systems to build capacity and enhance dissemination in heart health promotion: a Canadian multiple-case study. Health Educ Res 2005;20(5):499–513. | |
| OutPatient Treatment in Ontario Services (OPTIONS) Model | 5 | Martin GW, Herie MA, Turner BJ, Cunningham JA. A social marketing model for disseminating research-based treatments to addictions treatment providers. Addiction 1998;93(11):1703–15. | |
| ^1^ Number of times TMF is cited is from the “D&I Models in Health Research and Practice” website. | | | |

| **Supplemental Table S2.** Abbreviated version of the Covidence data extraction tool. The left column shows the variable name of the information extracted from the articles, the middle column shows the instructional prompt that was presented to the reviewer for this item, and the right column shows the possible responses options for each item. | | |
| --- | --- | --- |
| **Extraction item** | **Prompt** | **Available Item Responses** |
| *General Information* | | |
| Covidence #/ Study ID | Covidence # associated with the study | Free text |
| Country | Country(s) where the study took place | Canada; Australia; Netherlands; Norway; England/Great Britain; Ireland; South Korea; Singapore; China; New Zealand; Other |
| *Methods* | | |
| Focus area  of research | What is the focus area(s) of this research? | Rehabilitation; Physical Activity/Exercise; Mental Health (e.g., Depression, social connection); Patient Quality of Life; Quality of Clinical Care; Long Term Services and Supports; Other |
| Study  Design | What study design(s) were used? | Randomized Controlled Trial; Non-randomized experimental study; Cohort study, Cross sectional; Case control study; Qualitative research; Mixed-methods; Observational; Retrospective; Case report; Pre-post quasi-experimental; Pilot; Quality improvement; Administrative Data/ Database; Group; Measure development/Validation; Other |
| Participants/ Population Studied | Select the population(s) studied | Older adults; Caregivers; Staff/ Clinical Providers; Program; Community Collaborators; Other |
| Methods | What methods were used to measure the outcome(s) of the study? | Pre/Post survey; Administrative data; Focus groups; Interview; Change in clinical outcome; Survey/Questionnaire; Standardized clinical assessment (e.g., Timed up and Go); Other |
| Setting  Studied | What was the setting(s) or setting location of the population being studied? | Clinic; Hospital; Community-based program; Nursing Home; Non-institutional long-term services and supports; Other |
| Stage of implementation | What were the implementation stage(s) of the study, as described by the authors. If an intervention is adapted, please also select “Other” and provide information | Pre-implementation; Implementation; Sustainment; and Intervention adaptation (expand in other); Other |
| *TMF Information* | | |
| TMF from D&I website | Which TMF(s) were used in this article identified from the D&I website? | See list of TMFs in Table 2 |
| Additional TMFs | Select any additional TMF(s) that appear in the article that are NOT listed. |  |
| *Evaluation of TMFs* | | |
| TMF  Use | How was the TMF used in the article? | Introduction; Methods; Results; Discussion/Conclusion; Other |
| TMF  Diffusion | How many sections did the TMF appear | 1- once; 2- more than one section, but not all;  3- all sections of the article |
| Meaningful use of TMF | Meaningful use of cited TMF defined as guiding “data collection, measurement, coding, analysis, and/or reporting” | 0- Absent  1- Present |

**Supplemental Table S3:** Definitions and context for extracted variables in the data extraction form.

This document provides expanded definitions and contextual explanations for the variable items included in the data extraction form (see Supplemental Table S2). These definitions supported standardized data extraction and interpretation across reviewers.

| **Category** | **Definition** |
| --- | --- |
| *Focus Area of Research* | |
| Rehabilitation | Studies examining services such as physical therapy (PT), occupational therapy (OT), speech-language pathology (SLP), or physical medicine and rehabilitation (PM&R). This includes evaluations of interventions aligned with these disciplines. |
| Physical Activity/Exercise | Studies aiming to increase activity levels and/or general functional movements, either through structured exercise or general activity promotion. Interventions may be delivered by clinical providers and/or target older adults directly. |
| Mental Health | Studies that directly or indirectly address psychological well-being, including depression, anxiety, social isolation, or related indicators. |
| Quality of Clinical Care | Studies that seek to improve clinical decision-making or service delivery, including prescribing practices, clinical protocols, or safety/quality improvement initiatives. |
| Long-Term Services and Supports (LTSS) | Studies involving interventions delivered in an institutional (e.g., nursing homes, assisted living) or non-institutional (e.g., home-based, adult day health) setting(s). This category includes delivering or receiving medical and non-medical services such as personal care, advanced care planning, home health, hospice, and palliative care. |
| *Study Design* | |
| Process or Implementation Evaluation | Studies assessing the implementation processes associated with delivering interventions. These typically address dimensions such as reach, fidelity, barriers/facilitators, and contextual factors. Process evaluations are often embedded within broader trial designs (e.g., RCTs or mixed-methods research). |
| Group-Level Design | Applied when units of analysis were clustered (e.g., wards, organizations, municipalities). |
| *Setting Studied* | |
| Clinic | Typically, outpatient sites delivering health care, including primary care doctors offices, specialty outpatient services, or allied health clinics. |
| Hospital | Inpatient acute care or rehabilitation hospital settings. |
| Community-Based Programs | Programs operating in community centers, wellness facilities, or outreach organizations. |
| Nursing Homes | Licensed long-term care facilities. |
| Non-Institutional LTSS | Home and community-based services including care provided by home health aides, family caregivers, or within supportive housing models. |
| *Participants/Populations studied* | |
| Older Adults | Individuals aged ≥60 years or the mean age of the total sample was ≥60 years. |
| Staff/Clinical Providers | Health professionals, administrative staff, or paraprofessionals. |
| Caregivers | Family or informal caregivers. |
| Program | Refers to interventions, implementation teams, or delivery models evaluated as units. |
| *Implementation Stage and Intervention Adaptation* | |
| Pre-Implementation | Refers to activities such as contextual assessment, stakeholder engagement, or selection of evidence-based practices prior to active delivery. |
| Implementation | Involves active delivery of an intervention or program. |
| Sustainment | Refers to the continuation or institutionalization of an intervention beyond initial implementation, including studies evaluating long-term integration or ongoing outcomes. |
| Intervention Adaptation | Includes any tailoring or modification of an existing intervention for a new context, setting, or population. Adaptation frameworks (e.g., FRAME, FRAME-IS, Adaptome) may be referenced or inferred. |

| **Supplemental Table S4:** Included articles that cited a combination of more than one TMF. Columns one through five show the name of the first, second, third, fourth, and fifth TMF(s) cited. Column 6 shows the count of the times each combination was cited. Column 7 shows the count of how frequently the combination was rated as meaningful use. | | | | | | |
| --- | --- | --- | --- | --- | --- | --- |
| **TMF 1** | **TMF 2** | **TMF 3** | **TMF 4** | **TMF 5** | **Count** | **Count (%) of Meaningful Use*** |
| BCW^†^ | PARIHS |  |  |  | 2 | 0 (0) |
| BCW^†^ | Greenhalgh Diffusion of Innovation^†^ |  |  |  | 1 | 1 (100) |
| BCW^†^ | Knowledge Action Model^†^ |  |  |  | 4 | 2 (50) |
| BCW^‡^ | TDF |  |  |  | 3 | 1 (33) |
| BCW | NPT |  |  |  | 1 | 0 (0) |
| BCW^†^ | Explain Bx Change in EBP^‡^ |  |  |  | 6 | 3 (50) |
| BCW | Candelonian Practice |  |  |  | 1 | 1 (100) |
| CFIR | PARIHS |  |  |  | 2 | 0 (0) |
| CFIR^†^ | RE-AIM 1.0^‡^ |  |  |  | 2 | 0 (0) |
| CFIR | Greenhalgh Diffusion of Innovation |  |  |  | 3 | 0 (0) |
| CFIR | Knowledge Action Model |  |  |  | 1 | 0 (0) |
| CFIR | NPT |  |  |  | 1 | 0 (0) |
| CFIR† | Explain Bx Change in EBP |  |  |  | 1 | 0 (0) |
| CFIR | Proctors Imp Outcomes^‡^ |  |  |  | 2 | 0 (0) |
| CFIR | Dynamic Sustainability Framework |  |  |  | 1 | 1 (100) |
| CFIR | Weiner Organizational Readiness |  |  |  | 1 | 0 (0) |
| PARIHS^†^ | Greenhalgh Diffusion of Innovation |  |  |  | 1 | 0 (0) |
| PARIHS | Knowledge Action Model† |  |  |  | 1 | 0 (0) |
| PARIHS | Iowa Model of EBP |  |  |  | 1 | 0 (0) |
| PARIHS | Stetler Model of Research Utilization |  |  |  | 1 | 0 (0) |
| RE-AIM 1.0 | Explain Bx Change in EBP |  |  |  | 1 | 0 (0) |
| RE-AIM 1.0 | REP^†^ |  |  |  | 1 | 0 (0) |
| RE-AIM 1.0 | PRISM |  |  |  | 1 | 0 (0) |
| RE-AIM 1.0 | RE-AIM 2.0 |  |  |  | 1 | 1 (100) |
| Greenhalgh Diffusion of Innovation | NPT^†^ |  |  |  | 1 | 0 (0) |
| Greenhalgh Diffusion of Innovation | Proctors Imp Outcome |  |  |  | 1 | 0 (0) |
| Greenhalgh Diffusion of Innovation | REP |  |  |  | 1 | 0 (0) |
| Greenhalgh Diffusion of Innovation | General Theory of Implementation |  |  |  | 1 | 0 (0) |
| Greenhalgh Diffusion of Innovation | Determinants of Innovation |  |  |  | 2 | 0 (0) |
| Greenhalgh Diffusion of Innovation | Framework for Spread^†^ |  |  |  | 1 | 0 (0) |
| Knowledge Action Model | Organizational Theory of Innovation Implementation† |  |  |  | 1 | 0 (0) |
| Knowledge Action Model | Utilization Focused Surveillance |  |  |  | 1 | 0 (0) |
| Knowledge Action Model | Interactive Systems Framework |  |  |  | 1 | 1 (100) |
| Knowledge Action Model | CRARIUM† |  |  |  | 1 | 0 (0) |
| TDF^†^ | Explain Bx Change in EBP |  |  |  | 4 | 3 (75) |
| NPT | Explain Bx Change in EBP |  |  |  | 1 | 1 (100) |
| Explain Bx Change in EBP | Proctors Imp Outcomes^†^ |  |  |  | 1 | 0 (0) |
| REP | Dynamic Sustainability Framework |  |  |  | 1 | 0 (0) |
| Proctors Imp Outcomes | Conceptual Model of Imp Research |  |  |  | 1 | 1 (100) |
| BCW^†^ | Explain Bx Change in EBP | Intervention Mapping^†^ |  |  | 1 | 0 (0) |
| BCW | CFIR | Greenhalgh Diffusion of Innovation |  |  | 1 | 0 (0) |
| BCW† | CFIR | TDF |  |  | 1 | 0 (0) |
| BCW | CFIR^†^ | Health Equity Implementation Framework |  |  | 1 | 0 (0) |
| BCW | TDF | Explain Bx Change in EBP |  |  | 1 | 1 (100) |
| CFIR | PARIHS | RE-AIM 1.0 |  |  | 1 | 0 (0) |
| CFIR^†^ | PARIHS | Greenhalgh Diffusion of Innovation |  |  | 2 | 0 (0) |
| CFIR | PARIHS^†^ | Organizational Theory of Innovation Implementation |  |  | 1 | 0 (0) |
| CFIR | RE-AIM 1.0 | Greenhalgh Diffusion of Innovation |  |  | 1 | 0 (0) |
| CFIR | Greenhalgh Diffusion of Innovation | NPT^†^ |  |  | 2 | 0 (0) |
| CFIR | CBPR | Intervention Mapping^†^ |  |  | 1 | 0 (0) |
| CFIR | Explain Bx Change in EBP | Proctors Imp Outcomes |  |  | 1 | 0 (0) |
| CFIR | Determinants of Innovation^†^ | Generic Implementation Framework |  |  | 1 | 0 (0) |
| CFIR | Intervention Mapping | Stirman Framework for Adaptations |  |  | 1 | 0 (0) |
| PARIHS | Greenhalgh Diffusion of Innovation | NPT |  |  | 2 | 0 (0) |
| PARIHS^†^ | Greenhalgh Diffusion of Innovation | Framework for Dissemination and Utilization of Research |  |  | 1 | 0 (0) |
| PARIHS^†^ | NPT | Dynamic Sustainability Framework |  |  | 1 | 0 (0) |
| CFIR† | PARIHS^†^ | RE-AIM 1.0 | Dynamic Sustainability Framework |  | 1 | 0 (0) |
| CFIR | PARIHS | REP | Interactive Systems Framework |  | 1 | 0 (0) |
| CFIR | Greenhalgh Diffusion of Innovation | NPT^†^ | Dynamic Sustainability Framework |  | 1 | 0 (0) |
| CFIR† | Greenhalgh Diffusion of Innovation | Proctors Imp Outcomes^†^ | EPIS |  | 1 | 0 (0) |
| CFIR | Greenhalgh Diffusion of Innovation | Interactive Systems Framework | Stirman Framework for Adaptation |  | 1 | 0 (0) |
| PARIHS | Greenhalgh Diffusion of Innovation | Proctors Imp Outcomes | Determinants of Innovation |  | 1 | 0 (0) |
| Greenhalgh Diffusion of Innovation | Conceptual Model of Imp Research | Determinants of Innovation^†^ | Interactive Systems Framework |  | 1 | 0 (0) |
| RE-AIM 1.0 | Knowledge Action Model | Conceptual Model of Imp Research | Interactive Systems Framework^†^ | Dynamic Sustainability Framework | 1 | 0 (0) |
| **TOTALS** | | | | | **87** | **17 (20)** |
| ***** Meaningful use is defined as guiding data collection, measurement, coding, analysis, and/or reporting  † One TMF in the combination was used meaningfully  ‡ Two TMFs in the combination were used meaningfully | | | | | | |
| BCW = Behavior Change Wheel; CBPR = Community- Based Participatory Research; CFIR = Consolidated Framework for Implementation Research; Determinants of Innovation = Determinants of Innovation in Health Care Organizations; EPIS = Exploration, Preparation, Implementation, Sustainment; Explain Bx Change in EBP = Explaining Behavior Change in Evidence- Based Research; Framework for Dissemination and Utilization = Framework for Dissemination and Utilization of Research for Health-Care Policy and Practice; TDF = Theoretical Domains Framework; NPT = Normalization Process Theory; Proctors’ Imp Outcomes = Proctors’ Implementation Outcomes; PARIHS = Promoting Action on Research Implementation in Health Services PRISM = Practical, Robust, Implementation and Sustainability, Model; REP = Replicating Effective Programs | | | | | | |

| **Supplemental Table S5:** Categories of TMFs cited in international aging research | |
| --- | --- |
| **Name of theory, model, or framework** | **Model Categorization** |
| Behaviour Change Wheel | Strategy Framework |
| Consolidated Framework for Implementation Research | Determinant Framework |
| Promoting Action on Research Implementation in Health Services (PARIHS) | Determinant Framework |
| RE-AIM 1.0 Framework | Evaluation Framework |
| Explaining Behavior Change in Evidence-Based Practice * | Strategy Framework |
| Greenhalgh Diffusion of Innovations in Service Organizations | Determinant Framework |
| Normalization Process Theory | Implementation Theory |
| Pragmatic-Explanatory Continuum Indicator Summary 2 (PRECIS-2) * | Strategy Framework |
| Proctor's Implementation Outcomes | Evaluation framework |
| Canadian Institutes of Health Research Knowledge Translation within the Research Cycle Model or Knowledge Action Model (KTA) | Process Model |
| Theoretical Domains Framework | Determinant Framework |
| Interactive Systems Framework (ISF) | Determinant Framework |
| Dynamic Sustainability Framework | Determinant Framework |
| Intervention Mapping * | Strategy Framework |
| General theory of implementation | Determinant Framework |
| Determinants of Innovation within Health Care Organizations * | Determinant Framework |
| Stirman framework and coding system for modifications and adaptations of evidence-based interventions | Evaluation Framework |
| Davis' Pathman-PRECEED Model * | Process Model |
| Community Based Participatory Research (CBPR) * | Process Model |
| Replicating Effective Programs Framework * | Process Model |
| Pronovost's 4E's Process Theory | Process Model |
| Utilization-Focused Surveillance Framework * | Process Model |
| Conceptual Model of Implementation Research | Strategy Framework |
| Research Knowledge Infrastructure * | Strategy Framework |
| Exploration, Preparation, Implementation, Sustainment (EPIS) model | Determinant Framework |
| Determinants of Innovation within Health Care Organizations Service Organizations * | Determinant Framework |
| Practical, Robust Implementation and Sustainability Model (PRISM) | Determinant Framework |
| Weiner organizational readiness | Implementation Theory |
| Knowledge Exchange Framework * | Process Model |
| RE-AIM 2.0/Contextually Expanded RE-AIM * | Evaluation Framework |
| Critical Realism & the Arts Research Utilization Model (CRARIUM) * | Process Model |
| Knowledge Transfer and Exchange * | Process Model |
| Generic Implementation Framework * | Implementation Theory |
| Framework for Analyzing Adoption of Complex Health Innovations | Determinant Framework |
| Health Equity Implementation Framework | Determinant Framework |
| Policy Framework for Increasing Diffusion of Evidence-based Physical Activity Interventions * | Implementation Theory |
| Iowa Model of Evidence-Based Practice | Process Model |
| Designing and evaluating interventions to eliminate racial and ethnic disparities in health care * | Process Model |
| Stetler Model of Research Utilization | Process Model |
| "4E" Framework for Knowledge Dissemination and Utilization * | Process Model |
| Framework for Enhancing the Value of Research for Dissemination and Implementation (Framework for enhancing the value of D&I Research- reporting guidelines) | Strategy Framework |
| Ottawa Model of Research Use (OMRU) | Process Model |
| Evidence Integration Triangle * | Determinant Framework |
| Framework for the Dissemination & Utilization of Research for Health-Care Policy & Practice * | Determinant Framework |
| Caledonian Practice Development Model * | Strategy Framework |
| EMTReK - Evidence-based Model for the Transfer and Exchange of Research Knowledge * | Determinant Framework |
| Stages of Research Utilization Model | Process Model |
| Framework for Spread * | Determinant Framework |
| Organizational Theory of Implementation * | Implementation Theory |
| * TMF not in review by Wang et. al., 2024 | |

**
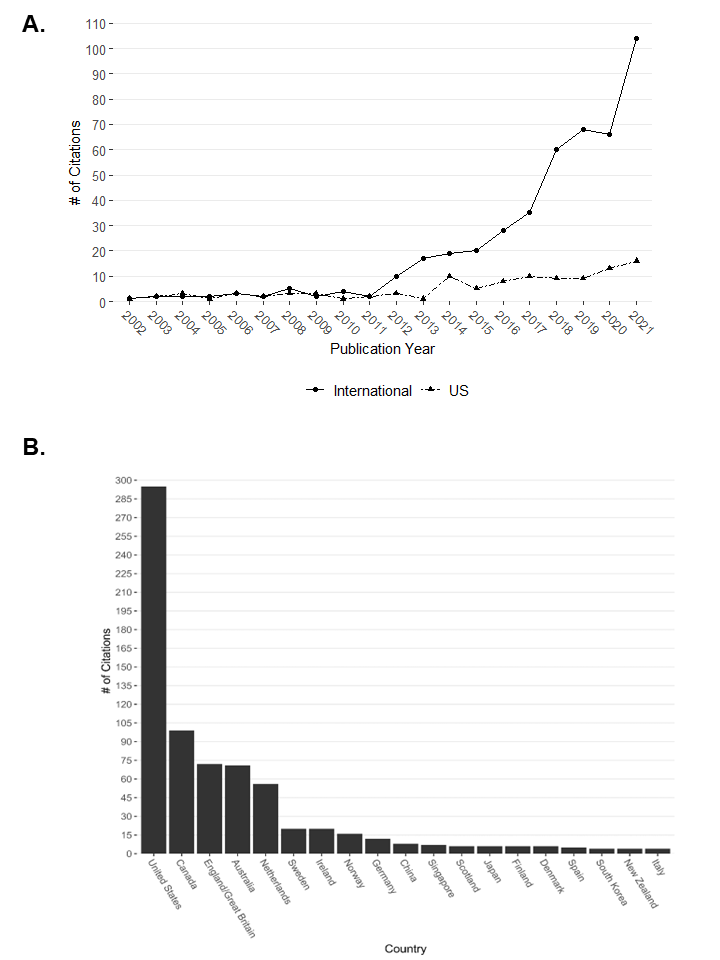
**

**Supplemental Fig 1A.** Line graph showing the trend of article count by year from 2002 to 2021 in 1-year increments for international countries (solid line) and the US (dashed line). US results are from our previous review.(Sullivan et al., 2022)

**Fig 1B.** Bar graph showing the count of articles by country for countries with at least 3 articles, including the US, in descending order. The US counts come from our previous review (Sullivan et al., 2022). The bars are presented in descending order from the country with the most articles, the United States (US) with ~295 articles. There is a sharp decrease to the second highest bar (Canada, ~90 articles) with a gradual decrease in article counts across remaining countries. The last several countries on the figure have very few articles (<5).

**References:** Sullivan, J. L., Montano, A. L., Hughes, J. M., Davila, H. W., O’Malley, K. A., Engle, R. L., Hawley, C. E., Shin, M. H., Smith, J. G., & Pimentel, C. B. (2022). A Citation Review of 83 Dissemination and Implementation Theories, Models, or Frameworks Utilized in U.S.-Based Aging Research. *The Gerontologist*. <https://doi.org/10.1093/geront/gnac096>
